# Supplementary material for: A simulation framework to determine optimal strength training and musculoskeletal geometry for sprinting and distance running
Source: PLoS Comput Biol. 2024 Feb 23;20(2):e1011410. doi: 10.1371/journal.pcbi.1011410 (PMC10917303; doi:10.1371/journal.pcbi.1011410)
Supplement: S1 Text — (DOCX) [file pcbi.1011410.s001.docx]

# SUPPLEMENTARY MATERIALS

Comparing predicted kinematics to experimental kinematics

Our simulated kinematics represent many of the key features of experimentally measured kinematics during running and sprinting. For marathon running, which we defined as running at 3.33m/s, we compared simulated kinematics and ground reaction forces to experimental data from ten individuals running at about 3.1m/s [1] (Figure A, B). The ankle, hip flexion, and hip adduction kinematics are similar. Our simulated hip rotation kinematics fall within the variability observed in experimental hip rotation kinematics. The knee kinematics have a similar waveform, but simulations show lower peak knee flexion during swing and greater knee flexion during stance. The pelvis, except for the rotation, and lumbar degrees of freedom have similar waveforms, but in the simulations there is a more limited range of motion. The pelvis posterior tilt is lower in simulation compared to the experimental data. This explains a similar but opposite offset in the lumbar extension angle and hip flexion angle, meaning that segment orientations for the torso and femurs do not have an offset between experiments and simulation.
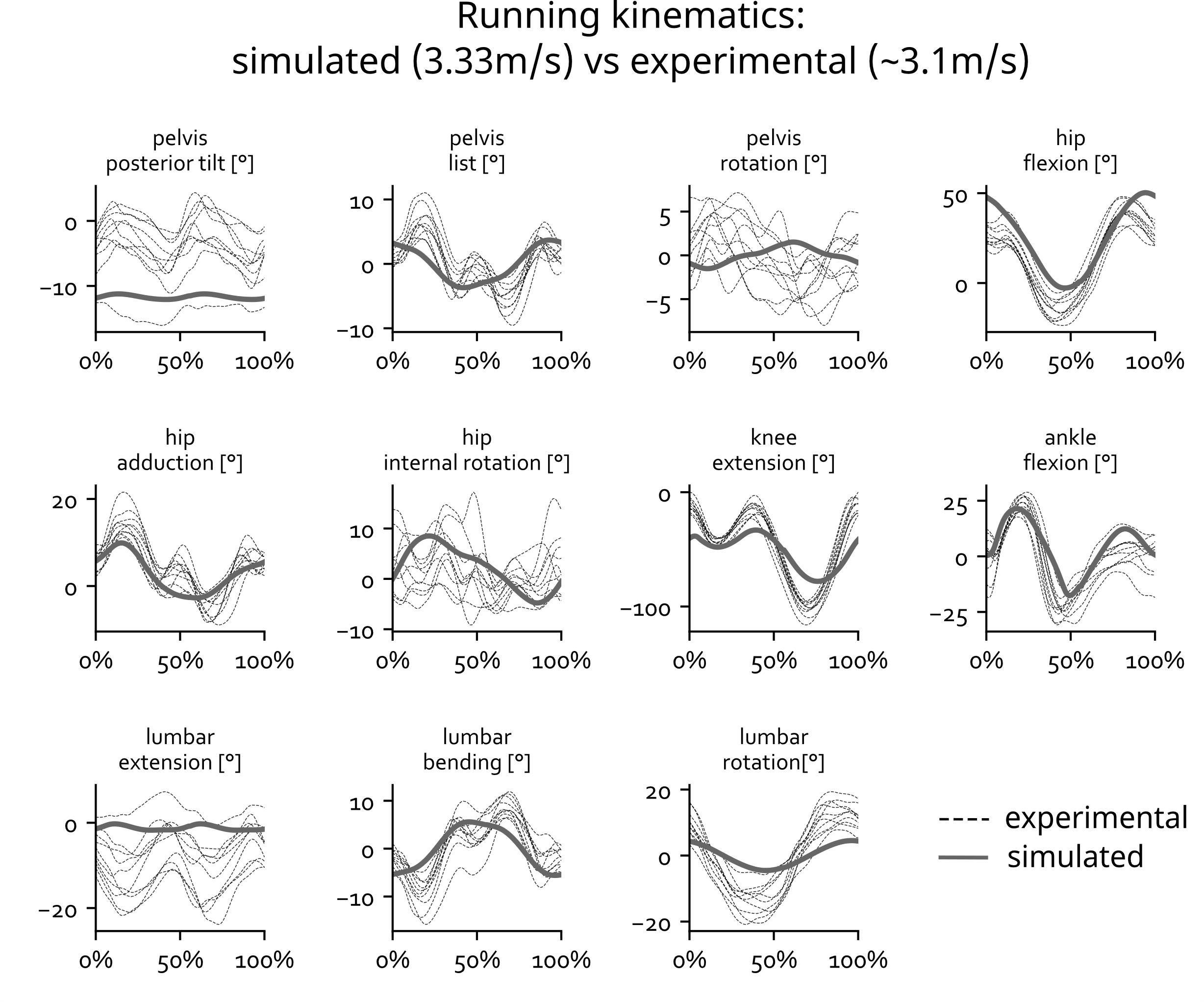


Figure A - Simulated (solid line) and experimental (dotted lines) kinematics during running. The gait cycle is defined from foot strike to foot strike.

Simulated and experimental ground reaction forces were also similar (Figure B). In our simulations the stance phase was slightly longer than in the experiments.


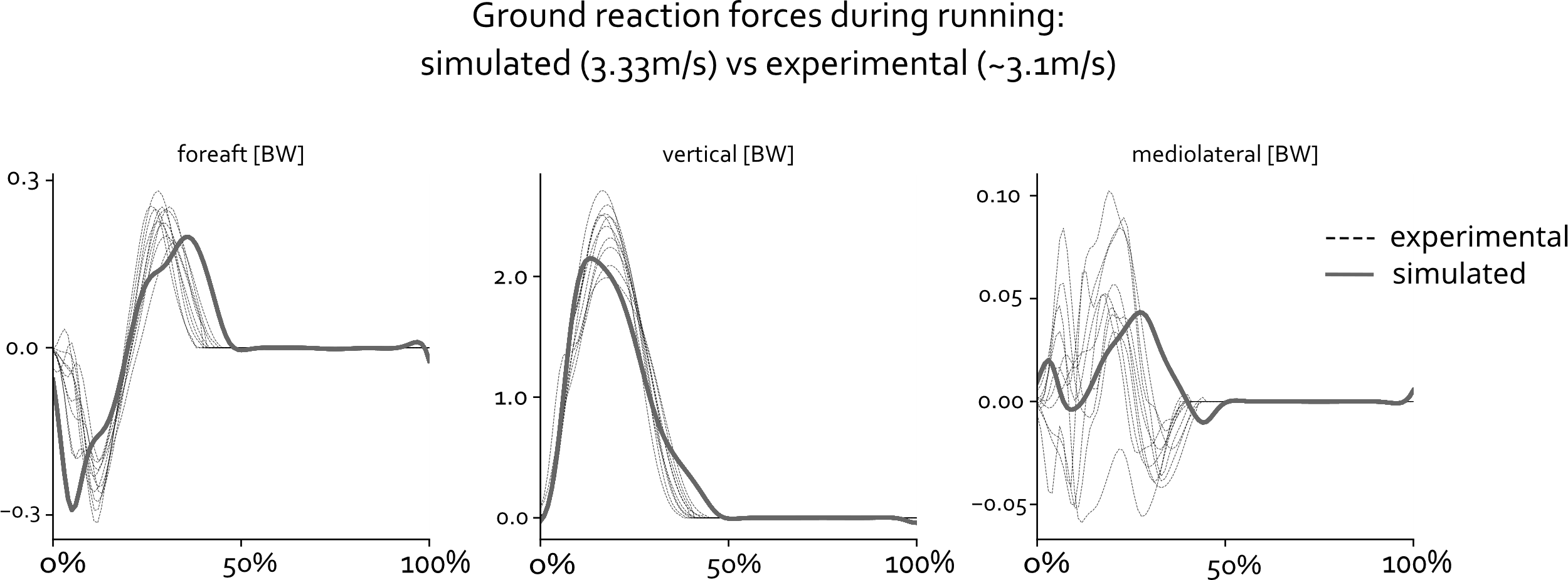


Figure B - Simulated (solid line) and experimental (dotted lines) ground reaction forces during running. The gait cycle is defined from foot strike to foot strike.

We compared our sprinting simulation (8.13m/s) to a sprinting gait cycle from an individual running at about 9.45m/s [2] (Figure C). The most important differences between simulated and experimental kinematics are a lower simulated peak knee and hip flexion during swing. This has been observed in other simulations studies as well [3]. These differences may be due to the fact that (1) we did not tune any of the muscle parameters (e.g., to reduce passive forces), which might prevent such high values for knee and hip flexion [4], and (2) our model has a lower sprint speed than in the experiment.

The shape of the ground reaction force and duration of contact are similar in simulation and the experiment (Figure D). The simulated vertical ground reaction force reaches a significantly higher peak than observed in the experiment. The contact impulse is similar for experiment and simulation (not shown explicitly) as the simulated higher peak is shorter than the experimental one. Such simulated running technique might be painful in reality because of high impact forces and therefore not seen in the experiment. Numerical parameters of contact models to represent real foot-ground contact are hard to determine. The parameters we used were calibrated against experimental data in a prior study [5], but due to the highly non-linear characteristics of contact physics large changes can occur when the model behaves outside of the experimental data for which it was calibrated. The fore-aft ground reaction force is smaller in simulation for both the braking and acceleration component.


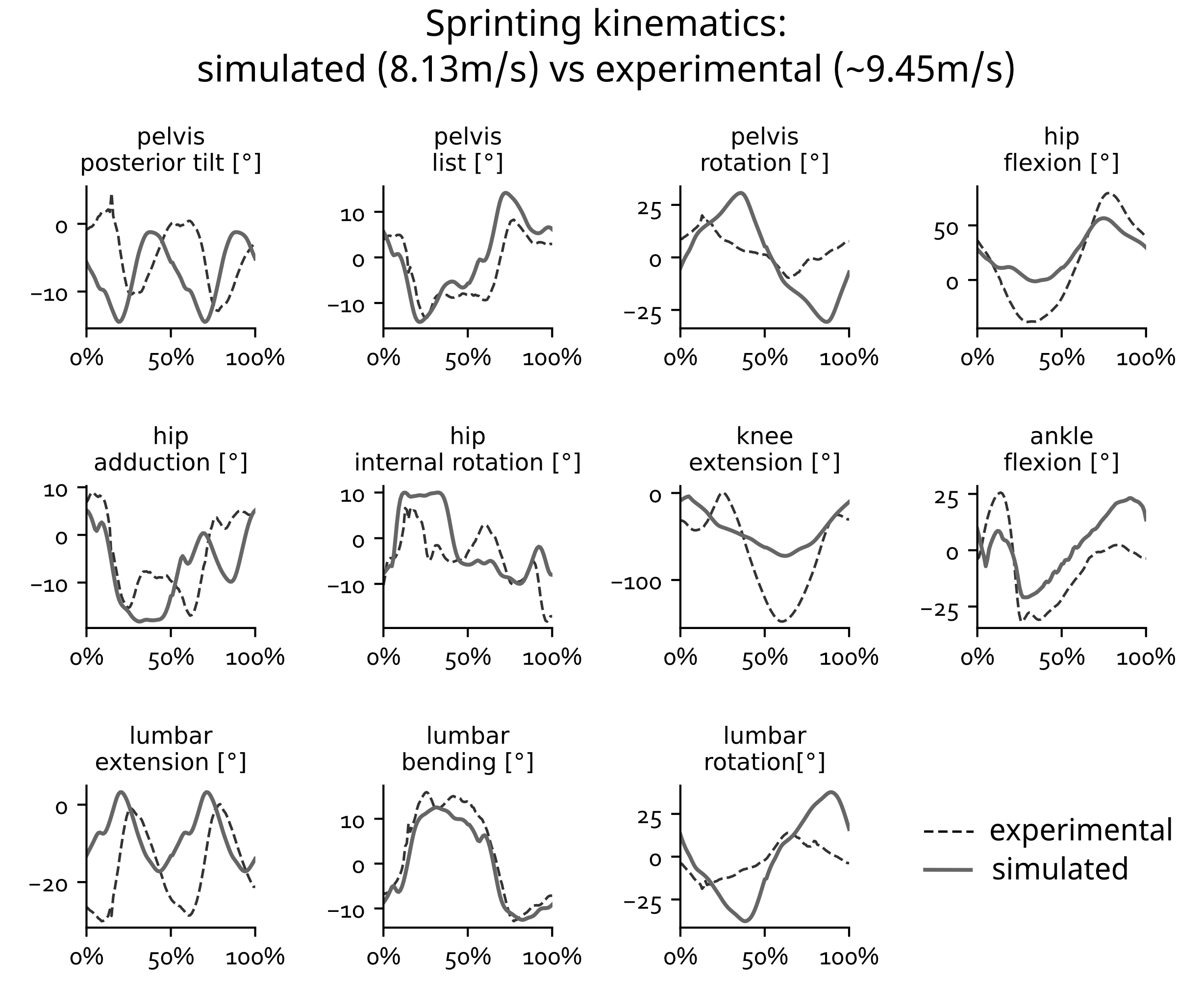


Figure C - Simulated (solid line) and experimental (dotted lines) kinematics during sprinting. The gait cycle is defined from foot strike to foot strike.


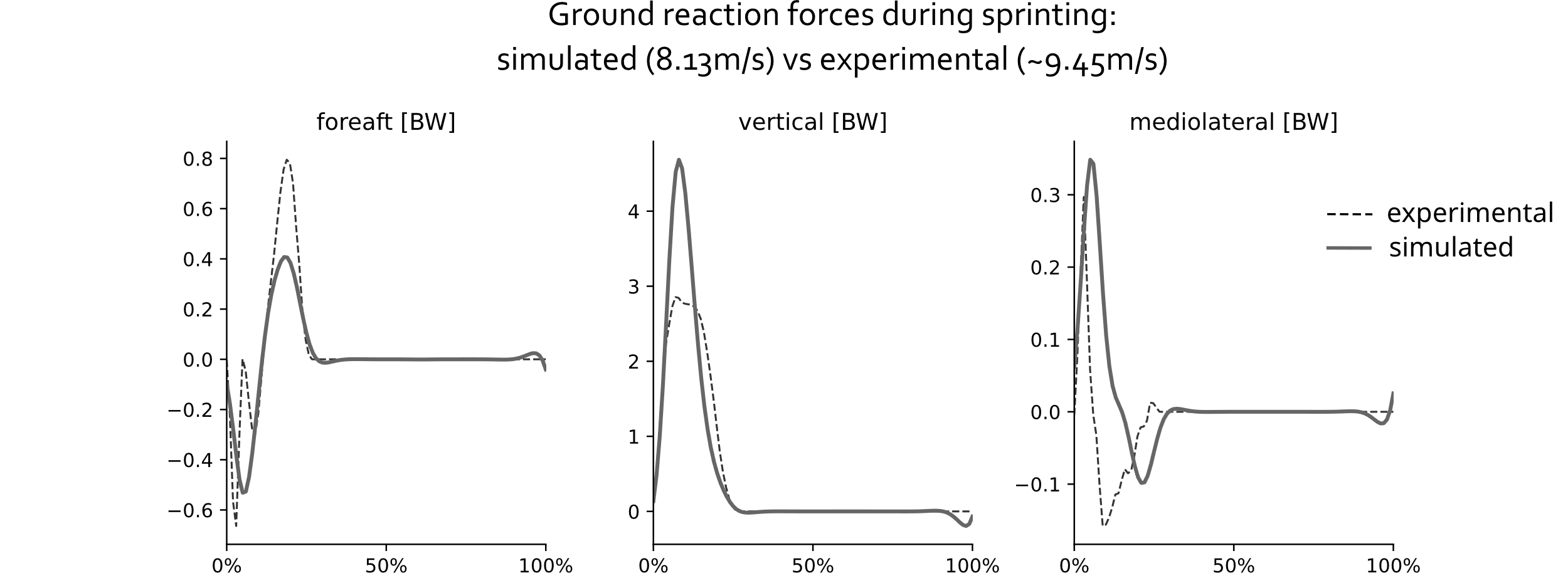


Figure D - Simulated (solid line) and experimental (dotted lines) ground reaction forces during sprinting. The gait cycle is defined from foot strike to foot strike.

Validating muscle-tendon length moment arm and approximation

Our neural network approximates the muscle-tendon lengths and moment arms within 2mm for all poses during a sprinting cycle and across all optimized models (Figure E). For each model, we calculated for each muscle the average absolute error between ground truth and our approximation of muscle-tendon length and moment arm during sprinting. The mean errors are well below 1mm.


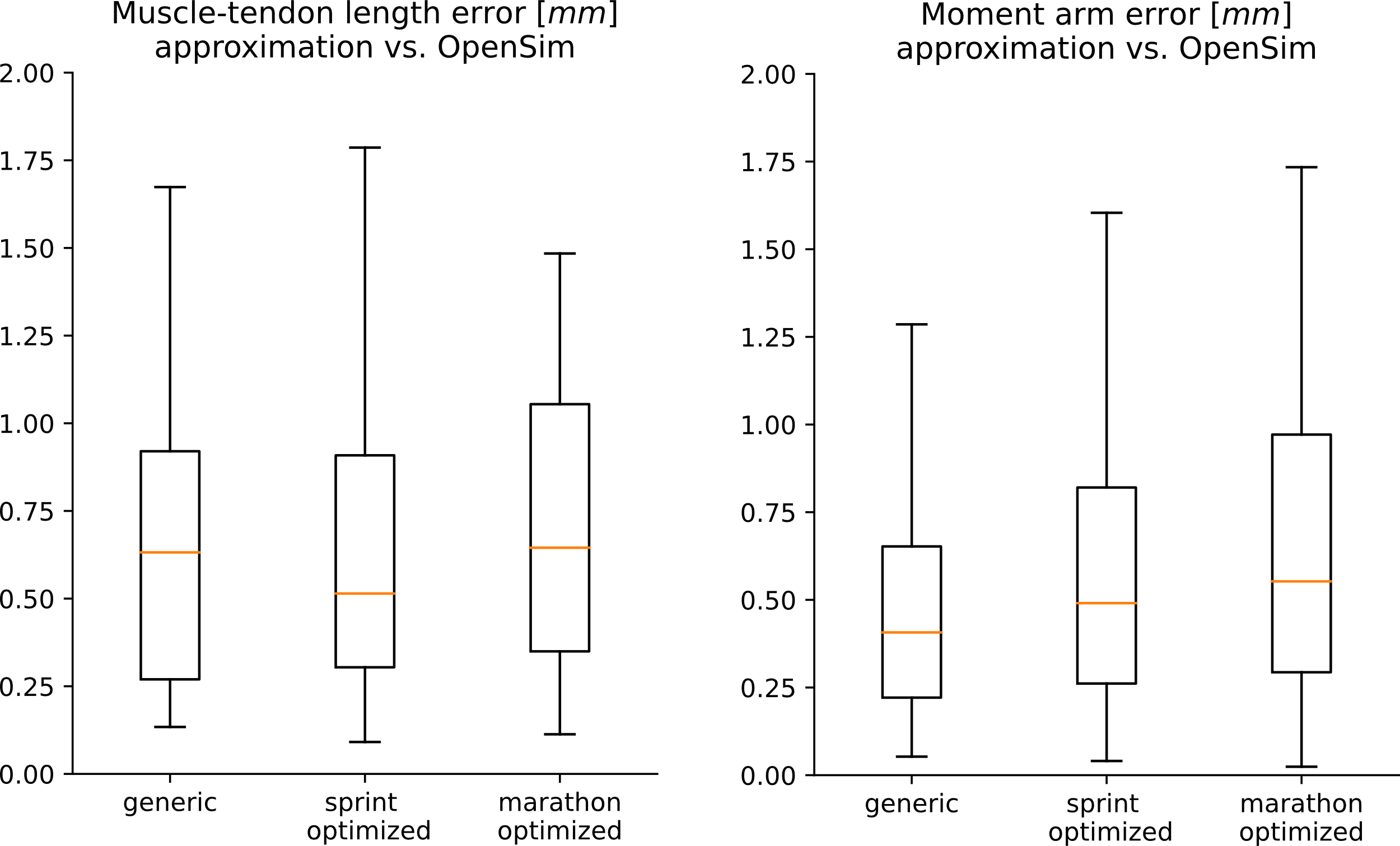


Figure E - Muscle-tendon length and moment arm error. We show average absolute errors across all the muscles.

1. Hamner SR, Seth A, Delp SL. Muscle contributions to propulsion and support during running. J Biomech. 2010;43: 2709–2716. doi:10.1016/J.JBIOMECH.2010.06.025

2. Dorn TW, Schache AG, Pandy MG. Muscular strategy shift in human running: dependence of running speed on hip and ankle muscle performance. J Exp Biol. 2012;215: 1944–1956. doi:10.1242/JEB.064527

3. Lin YC, Pandy MG. Predictive Simulations of Human Sprinting: Effects of Muscle-Tendon Properties on Sprint Performance. Med Sci Sports Exerc. 2022;54: 1961–1972. doi:10.1249/MSS.0000000000002978

4. Catelli DS, Wesseling M, Jonkers I, Lamontagne M. A musculoskeletal model customized for squatting task. Comput Methods Biomech Biomed Engin. 2019;22: 21–24. doi:10.1080/10255842.2018.1523396/SUPPL_FILE/GCMB_A_1523396_SM7370.DOCX

5. Falisse A, Afschrift M, De Groote F. Modeling toes contributes to realistic stance knee mechanics in three-dimensional predictive simulations of walking. PLoS One. 2022;17: e0256311. doi:10.1371/JOURNAL.PONE.0256311
