# Supplementary material for: A simulation framework to determine optimal strength training and musculoskeletal geometry for sprinting and distance running
Source: PLoS Comput Biol. 2024 Feb 23;20(2):e1011410. doi: 10.1371/journal.pcbi.1011410 (PMC10917303; doi:10.1371/journal.pcbi.1011410)
Supplement: Attachment — Submitted filename: PCOMPBIOL-D-23-01242_Review.pdf [file pcbi.1011410.s008.pdf]

Manuscript Review

Title

Simulated musculoskeletal optimization for sprinting and marathon running

This is a well-written article in which the authors developed a novel musculoskeletal simulator that is differentiable with respect to musculoskeletal geometry and muscle volumes. The differentiable simulator developed, which allows for considerable improvements in computational time of human movement optimizations, is a significant step forward in the field of musculoskeletal modeling and simulation. The authors demonstrate the utility of their newly developed simulator by implementing it to optimize body segment dimensions and muscle volumes for marathon running and sprinting. However, there are some issues and concerns that need to be addressed.

Major Comments

1. The absence of the discussion or consideration of differences in muscle fiber type between marathon runners and sprinters throughout the manuscript is a limitation, particularly given the known relationship between the proportion of type I and type II fibers and success in slower, longer distance events compared to higher velocity, shorter distance events.[a,b] Additional information should be provided with regards to the parameters included in the muscle models used in simulation that reflect fiber type (e.g., Af, activation & deactivation time constants, etc.). Moreover, a discussion of potential limitations and the effects of not accounting for fiber type different on the results of the simulations and the conclusions drawn from those results is warranted.

   References:

   a. Tesch P.A., Karlsson J. Muscle Fiber Types and Size in Trained and Untrained Muscles of Elite Athletes. *J. Appl. Physiol. (1985)* 1985;59:1716–1720. doi: 10.1152/jappl.1985.59.6.1716.
   b. Trappe S., Luden N., Minchev K., Raue U., Jemiolo B., Trappe T.A. Skeletal Muscle Signature of a Champion Sprint Runner. *J. Appl. Physiol. (1985)* 2015;118:1460–1466. doi: 10.1152/japplphysiol.00037.2015

2. The first paragraph of the methods state that "Importantly, when scaling the skeleton, we chose to scale muscle volumes proportionally to the change in whole body mass…" However, it is unclear what is meant by 'proportionally.' Is this a 1:1 relationship (e.g., whole body mass increased by 10% so total muscle volume increased by 10%), a 2:1 relationship (e.g., whole body mass increased by 10% so total muscle volume increased by 5%), some other proportion? Ultimately, I am trying to understand if there is an assumption that only muscle mass is increased with increased body mass or if there is some accounting for increased fat mass with increased body mass.
3. There appears to be a discrepancy between the optimal findings of the simulation and similar metrics for elite athletes, particularly for the step length and frequency in marathon running. Eliud Kipchoge's average cadence is on the range of 180-190 steps/minute with a step length of 1.9 meters, which is a considerable difference from the 0.94 step length and 210 steps/minute (3.5 Hz * 60 s/min) of the marathon simulation. Granted Kipchoge's speed is faster than 3.3 m/s, but it seems worth noting that this is done by increasing step length vs cadence. A discussion of

the discrepancies between the predicted optimal simulation and elite performance characteristics seems warranted, including commentary on what the model might not be capturing or assumptions that may be influencing this result.

4. In the results, the findings related to muscle volume are discussed in detail for the sprinting simulations, but not mentioned (though presented in Figure 2) for marathon running. This is a confusing omission based on the stated aims in the last paragraph of the Introduction.

Minor Comments

1. Abstract, 6th sentence: missing the word ‘as’ – “… in sprinters, as well as a mass in the …”
2. Introduction, 2nd paragraph 3rd to lass sentence: missing the word ‘of’ – “range of tasks”
5. Results/Supplementary Material: Ten simulations are noted in Table S1; however, it is unclear which results are associated with which simulations and whether results from all 10 simulations are discussed. It would be helpful to associate results with the simulations from which they were obtained.
3. Results, 3rd paragraph, 2nd sentence: Although the distance and speed are specified in the previous sentence for simulations of marathon running, it is unclear over what distance running speed was maximized for the sprinting simulations. I could not find this information in the methods either. I would expect the ability to accelerate (how quickly the model could reach maximum speed) would be an important factor in sprinting, and thus a confined distance over which to accelerate would be an important task constraint. Perhaps I am misunderstanding the sprinting simulation, in which greater detail is still needed to understand the imposed constraints.
4. Results, 5th paragraph, 2nd sentence: based on Figure 1, the increased pelvis width was only observed in the sprint-optimized model (not both models) as the sentence currently implies as written.
5. Methods, end of the paragraph which starts with “The skeleton parameters”: Is τ (via the R matrix) not also a quantity of skeleton dynamics (as defined in the equation for $\ddot{q}$ on the previous page) that is affected by the scaling of the body segments? While the moment arm matrix is mentioned in the following paragraph, it seems an odd omission from the list which includes the rest of the terms in the skeleton dynamics equation.
6. Methods, 2nd paragraph under “Differentiable musculoskeletal geometry computation,” 1st sentence: unnecessary parentheses in the 3rd line. Also, in the following sentence the word ‘are’ is missing: “Both parts are implemented”
7. Methods, 6th paragraph under “Differentiable musculoskeletal geometry computation,” last sentence: Please add the word ‘training’ before samples to improve clarity.
8. Methods, 7th paragraph under “Differentiable musculoskeletal geometry computation,” last sentence: There is a switch between “joint coordinates” in the previous paragraph to “generalized coordinates” in this paragraph. Consistent terminology would be preferrable for clarity.
