# Supplementary material for: A simulation framework to determine optimal strength training and musculoskeletal geometry for sprinting and distance running
Source: PLoS Comput Biol. 2024 Feb 23;20(2):e1011410. doi: 10.1371/journal.pcbi.1011410 (PMC10917303; doi:10.1371/journal.pcbi.1011410)
Supplement: Attachment — Submitted filename: Review_replies.docx [file pcbi.1011410.s009.docx]

We thank the reviewers for their effort and giving us very useful feedback that definitely improved our paper.

We included three reviewed versions of the article along with the replies in this document. ‘tracked changes’ contains the word document with all changes tracked in Word. Because this document can be confusing we added a colored version where we indicated text in yellow that is new or changed fundamentally (small changes and typos are not indicated). Finally, there is a ‘clean’ reviewed version.

In our replies we refer to lines where text is changed/added. These line references relate to the colored or clean version (not the tracked changes).

We also added several videos of the simulated motions in a .zip file.

**Reviewer 1**

This is a well-written article in which the authors developed a novel musculoskeletal simulator that is differentiable with respect to musculoskeletal geometry and muscle volumes. The differentiable simulator developed, which allows for considerable improvements in computational time of human movement optimizations, is a significant step forward in the field of musculoskeletal modeling and simulation. The authors demonstrate the utility of their newly developed simulator by implementing it to optimize body segment dimensions and muscle volumes for marathon running and sprinting. However, there are some issues and concerns that need to be addressed.

We appreciate the reviewers’ detailed evaluation and respond to each comment below.

**Major Comments**

1. The absence of the discussion or consideration of differences in muscle fiber type between marathon runners and sprinters throughout the manuscript is a limitation, particularly given the known relationship between the proportion of type I and type II fibers and success in slower, longer distance events compared to higher velocity, shorter distance events.^a,b^ Additional information should be provided with regards to the parameters included in the muscle models used in simulation that reflect fiber type (e.g., Af, activation & deactivation time constants, etc.). Moreover, a discussion of potential limitations and the effects of not accounting for fiber type different on the results of the simulations and the conclusions drawn from those results is warranted.

References:

a. Tesch P.A., Karlsson J. Muscle Fiber Types and Size in Trained and Untrained Muscles of Elite Athletes. J. Appl. Physiol. (1985) 1985;59:1716–1720. doi: 10.1152/jappl.1985.59.6.1716.

b. Trappe S., Luden N., Minchev K., Raue U., Jemiolo B., Trappe T.A. Skeletal Muscle Signature of a Champion Sprint Runner. J. Appl. Physiol. (1985) 2015;118:1460–1466. doi: 10.1152/japplphysiol.00037.2015

The reviewer points out an important physiological parameter that we have assumed to be identical and constant for all of our simulations. By keeping fiber type (contraction time constants) constant for all simulations, we were able to isolate the effects of musculoskeletal geometry and muscle mass distribution. However, we agree this would be an interesting and important extension to our work, and we highlight the limitation in the following new paragraph in the discussion:

There are many ways that a musculoskeletal model can be personalized to represent different individuals. For example, the researchers in past studies have scaled a generic OpenSim [37] model’s muscle volumes to simulate elite sprinting [35] and adapted musculoskeletal geometry for deep squats [63]. Additional muscle parameters that could be individualized and potentially contribute to performance in runners are the force-length, force-velocity and muscle activation times. These parameters vary according to muscle fiber type distribution, which is specialized in sprinters and distance runners [61,62]. In the current study, we chose to keep fiber type constant to isolate the effects of musculoskeletal geometry and muscle mass distribution.

We have also updated the Methods section to describe the activation and deactivation time constants (15ms and 60ms, respectively; lines 193), and the source of the force-length and force-velocity relationships (lines 205)

2. The first paragraph of the methods state that “Importantly, when scaling the skeleton, we chose to scale muscle volumes proportionally to the change in whole body mass…” However, it is unclear what is meant by ‘proportionally.’ Is this a 1:1 relationship (e.g., whole body mass increased by 10% so total muscle volume increased by 10%), a 2:1 relationship (e.g., whole body mass increased by 10% so total muscle volume increased by 5%), some other proportion? Ultimately, I am trying to understand if there is an assumption that only muscle mass is increased with increased body mass or if there is some accounting for increased fat mass with increased body mass.

We scaled with a 1:1 proportion, assuming a constant mass percentage of fat, muscle, bone, and other tissue as overall mass increased. We revised the manuscript to clarify this point (lines 145-146).

When scaling the skeleton, we chose to scale muscle volumes proportionally to the change in whole body mass with a 1:1 ratio. Individual body segments are scaled by assuming constant density. As such, the mass percentage of muscle and fat tissue remains constant for models of different sizes and thus a heavier model has stronger muscles.

3. There appears to be a discrepancy between the optimal findings of the simulation and similar metrics for elite athletes, particularly for the step length and frequency in marathon running. Eliud Kipchoge’s average cadence is on the range of 180-190 steps/minute with a step length of 1.9 meters, which is a considerable difference from the 0.94 step length and 210 steps/minute (3.5 Hz * 60 s/min) of the marathon simulation. Granted Kipchoge’s speed is faster than 3.3 m/s, but it seems worth noting that this is done by increasing step length vs cadence. A discussion of

the discrepancies between the predicted optimal simulation and elite performance characteristics seems warranted, including commentary on what the model might not be capturing or assumptions that may be influencing this result.

The reviewer points out an important discrepancy, which we now address in the following new paragraph of the discussion (lines 579-588):

Simulated marathon running had a step frequency (210 steps per minute) that is higher than what is typically observed in real-world sprinting (170 steps per minute) [70]. At a specific speed, a runners’ preferred step frequency coincides with the metabolic energy minimum [70],  and model errors that affect metabolic energy computation could explain the higher simulated step frequency. For example, the metabolic energy model used in our study might underestimate the increasing metabolic cost of producing (the same) cyclic force at increased rates. This phenomenon is described by Doke and Kuo [71] and also suggested by Swinnen et al. [72]. It is hypothesized to be associated with activation-deactivation dynamics, and the cost of calcium transport into the sarcoplasmic reticulum (SR) [73]. Next, the presence of an activation squared in our objective function might bias the model to run with higher cadence as it has been shown that some muscles operate at lower average activations at higher cadences [70].

4. In the results, the findings related to muscle volume are discussed in detail for the sprinting simulations, but not mentioned (though presented in Figure 2) for marathon running. This is a confusing omission based on the stated aims in the last paragraph of the Introduction.

The reviewer is correct that we only very briefly touch on the outcomes for the marathon-optimized strength training. Because the difference in performance was so small (0.3% improvement), an in-depth discussion could be confusing or distracting. We revised the manuscript to clarify (lines 571-578):

The small effects of strength training on marathon performance (0.3% improvement) indicate that strength training is less important for marathon runners than for sprinters. Therefore, we do not discuss the redistribution of muscle volume for this case in detail, and suggest that the results for the marathon-optimized model in figure 2.D should be interpreted with care.

**Minor Comments**

1. Abstract, 6th sentence: missing the word ‘as’ – “… in sprinters, as well as a mass in the …”

Thank you for catching this error. We have corrected this typo.

2. Introduction, 2nd paragraph 3rd to last sentence: missing the word ‘of’ – “range of tasks”

Thank you for catching this. We have corrected this typo.

5. Results/Supplementary Material: Ten simulations are noted in Table S1; however, it is unclear which results are associated with which simulations and whether results from all 10 simulations are discussed. It would be helpful to associate results with the simulations from which they were obtained.

We have updated the table to explicitly refer to the figures where these simulations have results displayed. The model names in the table also correspond to the legends/labels in the figures. The activity (sprinting/running) names in the table correspond to the activities shown in the figures. We also moved the table to the methods section, which now precedes the results.

3. Results, 3rd paragraph, 2nd sentence: Although the distance and speed are specified in the previous sentence for simulations of marathon running, it is unclear over what distance running speed was maximized for the sprinting simulations. I could not find this information in the methods either. I would expect the ability to accelerate (how quickly the model could reach maximum speed) would be an important factor in sprinting, and thus a confined distance over which to accelerate would be an important task constraint. Perhaps I am misunderstanding the sprinting simulation, in which greater detail is still needed to understand the imposed constraints.

We simulate only one gait cycle (technically half a gait cycle with a symmetry constraint). This is stated in the Methods section of the revised manuscript (lines 334-337).

As such the acceleration phase of sprinting is ignored. We acknowledge that this is an assumption that needs to be stated more clearly, so we added, in the beginning of the methods section (lines 137-139), more explicitly that we simulate the phase of running at maximal speed:

To simulate sprinting, running speed was maximized for a single simulated half gait cycle. We thus ignore the acceleration phase of sprinting and limit ourselves to simulating the maximal velocity.

4. Results, 5th paragraph, 2nd sentence: based on Figure 1, the increased pelvis width was only observed in the sprint-optimized model (not both models) as the sentence currently implies as written.

We have updated the sentence to clarify this point.

5. Methods, end of the paragraph which starts with “The skeleton parameters”: Is τ (via the R matrix) not also a quantity of skeleton dynamics (as defined in the equation for 𝑞𝑞̈ on the previous page) that is affected by the scaling of the body segments? While the moment arm matrix is mentioned in the following paragraph, it seems an odd omission from the list which includes the rest of the terms in the skeleton dynamics equation.

The reviewer is correct, and we added the joint torques as suggested (line 234).

6. Methods, 2nd paragraph under “Differentiable musculoskeletal geometry computation,” 1st sentence: unnecessary parentheses in the 3rd line. Also, in the following sentence the word ‘are’ is missing: “Both parts are implemented”

We have made these updates.

7. Methods, 6th paragraph under “Differentiable musculoskeletal geometry computation,” last sentence: Please add the word ‘training’ before samples to improve clarity.

We have made this update to improve clarity.

8. Methods, 7th paragraph under “Differentiable musculoskeletal geometry computation,” last sentence: There is a switch between “joint coordinates” in the previous paragraph to “generalized coordinates” in this paragraph. Consistent terminology would be preferrable for clarity.

We updated the manuscript to use joint coordinates when they refer to biological joints and use generalized coordinates (which include joint coordinates + root orientation and translation) when we need to describe all degrees of freedom.

**Reviewer 2**

The authors of this manuscript have developed a differentiable musculoskeletal simulator and used it to optimize body segment dimensions and muscle volumes for sprinting and marathon running. The technical contributions of this study are excellent but there are numerous weaknesses with the application to running. In both the technical and scientific aspects there are also numerous issues with the manuscript that impair clarity and negatively impact the potential of this paper in its current form. In my review below I have provided specific suggestions in an effort to help the authors address these concerns.

We thank the reviewer for the detailed suggestions to improve the manuscript; we have addressed this feedback, which has significantly improved the paper.

**General Comments:**

This manuscript does not provide page or line numbers, which makes it very cumbersome for the reviewer to provide specific comments on the paper. I strongly urge the authors to include page and line numbers in their future journal submissions. In places below where I refer to a page number I started counting from the page with the title, authors, and abstract as page 1.

We apologize for the lack of page and line numbers. We have added these to the revised manuscript.

The manuscript is replete with the word “higher” when there is no intent to refer to vertical position. This sometimes impairs the clarity of passages, and it can lead to some awkward phrases, such as “a higher relative lower” (Page 4). In most, if not all, of these cases it would be preferable to use a word like “greater” in place of “higher.”

Thank you for this feedback; we adapted our wording in several places by replacing “higher” with “greater” or “increased”.

The manuscript ignores some of the key literature that tries to determine factors affecting peak sprinting speed (e.g., page 11: Determinants of sprinting performance.) Addressing this literature would add depth and substance to the discussion section. See for example: Chapman & Caldwell, Journal of Biomechanics, 1983, Weyand et al., Journal of Applied Physiology, 2010, Miller et al., Journal of Biomechanics, 2012. The authors have also missed arguably the most closely related study to their own, in which muscle volume is optimized to maximize vertical jump height. (Wong et al., PLoS One. 2016). (e.g., relevant to the bottom of page 4, and possibly in the discussion section). Addressing these issues and other mentioned below would enhance the scientific merit of what is otherwise a sound study.

Thank you for pointing us to these references; we have incorporated these past studies into the introduction and discussion sections of the paper. We cited Wong et al. 2016 as prior work that uses more simplified musculoskeletal models (line 112). The other references are also relevant and we have discussed them as described below.

In Miller et al., the authors discuss two previously formulated mechanisms that could lead to reduced top sprinting speeds. We added a paragraph to discuss our results in the context of these theories (lines 535-545):

Two mechanisms have been proposed in previous work as limiters of maximal running speed: (1) decreasing ability to support body weight due to decreased stance time with increasing running speed [59,60] and (2) inability to quickly propel the limb forward during early swing, followed by energy removal in late swing to arrest forward motion and accurately position the foot [61]. Miller et al. [34] analyzed the effect of changing the force-velocity relationship on maximal running speed and analyzed whether changes in the force-velocity relationship affect running speed through these mechanisms. While Miller et al. found that both mechanisms could play a role, our simulations do not provide strong evidence for mechanism (1). Stance-phase hip extension torques did not increase at higher running speeds and although stance knee extension torques increased this did not require stronger vastii. The increases in hip flexion and extension torques at higher speeds along with stronger hip and knee flexor and hip extensor muscles in our optimizations provide support for mechanism (2).

**Specific Comments:**

Normally I favor brief titles. However, similar wording to the current title is often used to describe optimizing only the motion of a musculoskeletal model. By adding a few more words the authors could make it clear that they also optimized the structure of the musculoskeletal system, which might not be obvious to many potential readers when they first see the current title.

Original title: Simulated musculoskeletal optimization for sprinting and distance running

We changed the original title to:

A simulation framework to determine optimal strength training and musculoskeletal geometry for sprinting and distance running

.

Page 1: “However, our ability to understand how these parameters affect task performance has been limited due to the high computational cost of modelling the necessary complexity of the musculoskeletal system and solving the requisite multi-dimensional optimization problem.”

Comment: The wording could be improved because it assumes right away that the only way to study these matters is using a modeling approach.

We agree and  added a sentence just before this statement to describe limitations of experiments briefly (lines 12-16).

Musculoskeletal geometry and muscle volumes vary widely in the population and are intricately linked to the performance of tasks ranging from walking and running to jumping and sprinting. As an alternative to experimental approaches, where it is difficult to isolate factors and establish causal relationships, simulations can be used to independently vary musculoskeletal geometry and muscle volumes, and develop a fundamental understanding

Page 1: “Our simulation results can help sprint and marathon runners customize strength training”

Comment: This statement overreaches. No runner is going to read this one study and know how to change their training. This study can at best suggest things that can be tested experimentally for verification before being used by athletes and coaches.

We agree and have revised our sentence to read (lines 31-32):

Our simulation results provide insight to inspire future studies to examine optimal strength training.

Page 3: “Falisse et al. [8] enabled automatic differentiation, a faster alternative to finite differentiation, for musculoskeletal simulation…”

Comment: It is fine to mention the Falisse study here due to the relevance of the specific approach used in the present study. However, it comes across as if that study might have been the first to avoid the use of slow finite difference. In fact, there is a long history of people using collocation methods with exact derivatives to enable rapid musculoskeletal simulation, and this should at least be acknowledged in that passage. For example see Kaplan and Heegaard (J Biomechanics, 2001) and van den Bogert et al. (Procedia IUTAM, 2011).

We agree and have updated the introduction to better acknowledge the history of direct collocation methods (lines 65-66):

Automatic differentiation, a faster alternative to finite differences, has been applied to speed up musculoskeletal simulation (e.g. [8,9]). More recently, Falisse et al. [10] implemented a framework that is based on the commonly used OpenSim models and software [11].

Page 3: “To exercise this new simulator…”

Comment: Since this is a study about exercise (running) I suggest using a different word here to avoid confusion.

We have updated this sentence (line 72) to use the phrase “test and apply this new simulator.”

Page 4: “stronger hip muscles and a deeper pelvis”

Comment: Please clarify for the reader what a “deeper” pelvis means.

We updated this sentence to explain that by deeper pelvis we mean larger along the anteroposterior axis (line 95).

Page 4: Last paragraph that continues onto the top of page 5.

Comment: Given how little there is that addresses the running application in this paper, it may be worth mentioning the AnyBodyRun tool (https://www.anybodyrun.com) as it shares some of the same goals as this paper, while differing in many details. I am not suggesting AnyBodyRun is of comparable complexity to the present work, just that there is some overlap.

We  have added references to studies that analyze  sprinting: Miller et al. 2012, Chapman and Caldwell et al. 1983, Weyand et al. 2010. We understand that the AnyBodyRun tool can also be considered a simulator for studying aspects of running and sprinting but did not find a peer-reviewed publication (only a masters thesis: https://projekter.aau.dk/projekter/files/294397245/gr9217bedommelse.pdf) and thus could not to refer to it.

Pages 5-10: Results section.

Comment: There are long passages in the Results section describing the methods that were used. This makes the Results section much longer than it needs to be and makes the reader have to work to identify the findings among the descriptions of the methods. Please keep the methods in the Methods section. With the “methods at the end” structure of the manuscript if there is a need to cue the reader in certain places about the approach, keep it as brief as possible (e.g., a sentence or phrase).

We agree and have moved the Methods section before the results. We also restructured both the Methods section and the Results section to avoid repetition. We now start the methods section with an overview of the simulations we performed before going into the simulator details. This allowed us to remove the first section of the results. We also moved the first section of the original results (which discusses simulator performance and validity) towards the end of the results section.

Comment: It might be helpful for the readers to include a results figure that shows the optimally scaled models to help visualize overall difference in body dimensions, and possibly zoomed in to see effects on moment arms (though perhaps the latter is not possible with this approach).

We have appended videos of the different models sprinting and marathon running to help the reader appreciate the changes in segment dimensions and how that affects how the whole body looks performing the running tasks.

Page 6: “Simulated kinematics and ground reaction forces for running at 3.33m/s and sprinting with the generic model were similar to experimental values”

Comment: The statement “were similar to experimental values” is disingenuous. This one short phrase does not align with the more nuanced comparisons described in the Supplement, and even then, the results are not all similar. For example, the simulated pelvis tilt (different magnitude) and pelvis rotation (opposite phasing) are not similar to the experimental data.

The reviewer is right. We have updated the results section to include a more accurate and comprehensive description of how well our simulations align with experimental data. This description is in a new section on simulator performance and validity that we have added in the end of the results section (lines 470-475):

Our simulated kinematics represent many of the key features of experimentally measured kinematics during running and sprinting, giving confidence in the validity of the musculoskeletal model to address our study aims. However there are also some discrepancies: during swing, simulated hip and knee flexion angles were smaller than observed in experiments for both sprinting and marathon running. A detailed comparison is made in the Supplementary Materials (Figures S.1, S.2 for marathon running and Figures S.3, S.4 for sprinting).

Page 6: “2074 kcal for the marathon-optimized body-segment dimensions compared to 3267 kcal for the generic model”

Comment: I think these energy consumption values are in the right ballpark for the marathon. It would be helpful to provide some values from the literature for reference so the reader can understand if these predictions are accurate or not.

We added the following paragraph to help the reader interpret the validity of the predicted sprinting speed and metabolic energy expenditure compared to experimental data for running (lines 476-478):

Predicted marathon energy consumption for the generic model (3267kcal at 75.2kg) was similar to experimental values measured in 10 recreational marathon runners (2792 ± 235kcal at 72.3kg) [54]. Predicted maximal sprinting speeds were 33% slower than elite sprinting speeds.

Page 8: “The sprint-optimized and generic models sprinted with similar step lengths (1.92 m and 1.93 m, respectively), but the sprint-optimized model sprinted with higher step frequency (4.9 Hz vs 4.2 Hz). The marathon-optimized and generic models both ran at marathon pace with a 0.94 m step length and step frequency of 3.5Hz.”

Comment: Much as with the preceding energy consumption values, it would be helpful to provide a range of experimental values from the literature to help the reader understand how accurate these stride parameter predictions are.

Reviewer 1 had a similar comment, which we have addressed by comparing to literature values and addressing why these might differ from our simulated values. In particular, we added a paragraph in the discussion to elaborate on the difference in cadence at marathon pace (lines 579-588).

Simulated marathon running had a step frequency (210 steps per minute) that is higher than what is typically observed in real-world sprinting (170 steps per minute) [54]. At a specific speed, a runners’ preferred step frequency coincides with the metabolic energy minimum [54], and model errors that affect metabolic energy computation could explain the higher simulated step frequency. For example, the metabolic energy model used in our study might underestimate the increasing metabolic cost of producing (the same) cyclic force at increased rates. This phenomenon is described by Doke and Kuo [55] and substantiated by Swinnen et al.  [56]. In addition, the use of an activation squared term in our objective function might bias the model to run with higher cadence as it has been shown that some muscles operate at lower average activations at higher cadences [54].

Page 11 and 13: Marathon running economy

Comment: The present study can establish cause-and-effect in the sense that something is changed in the model and the effects on running performance can be readily determined. However, it is worth being a bit more circumspect as to how much this study reveals the “mechanisms” behind these effects. As one specific example, on page 11 it is stated that shank-to-thigh ratio, leg length and step length influence running economy, but then on page 13 when this is discussed there is the same level of informed speculation as one would expect in an observational study. There is a balance to be found regarding what is, and what is not, explained in this study.

Thank you for this feedback. We changed the wording in the referenced section (from page 13 of the original document, now lines 562-563) to be more circumspect:

Our simulation results indicate that longer legs, a high leg-length-to-height ratio and a high shank-to-thigh length ratio improve marathon economy, in line with experimental findings [25,27].

We have made similar changes on lines 31-32 in the abstract (“Our simulation results provide insight …”), in the discussion at lines 524-527 (“These higher torques with constant muscle force generating capacities could be explained by the interplay of several factors …”), and lines 499-501 (‘Our simulations indicate that increasing strength could improve sprinting performance…. “).

Page 12: “both models optimized for sprinting produced greater knee extension torques at midstance, but they did not require a significant increase in knee extension joint torque capacity to do so.”

Comment: This is another example where the “determinants” of sprint performance feels to have a bit been oversold. This is certainly an interesting finding, but can you explain *how* is greater knee extension torques being produced without a greater capacity to do so?

We agree describe is helpful for the reader. We updated this sentence as follows (lines 524-527):

These higher torques with constant muscle force-generating capacities could be explained by the interplay of several factors, including higher muscle activation levels, different kinematics resulting in larger moment arms, and the muscles operating at a more favorable section of the force-length and force-velocity curves.

Page 15: “For example, the simulator could determine minimal strength requirements to safely perform activities of daily living, could guide strength training interventions in elderly people, or estimate the effects of musculoskeletal surgery.”

Comment: It is a bit of a leap to go from identifying the largely (if not entirely) genetically determined factors that favor sprint and marathon running performance to ergonomic applications or predicting surgical outcomes. It might be helpful to take a few sentences to better explain future applications rather than try to cover it all within the final sentence of the Discussion section.

We agree that our original statement made a large logical leap and have added additional context to make our reasoning more clear (lines 644-650).

While the present study focused on athletic performance, our approach could also be used in other applications where muscle strength or musculoskeletal geometry affect mobility. For example, the simulator could determine minimal strength requirements to safely perform activities of daily living, guide strength training interventions in elderly people who struggle with specific activities of daily living, or help plan musculoskeletal surgery that aims to enable or improve the performance of specific activities by changing musculoskeletal geometry.

Page 15: “The third and fourth simulations”

Comment: After this statement the authors stop counting out the simulations for the reader. It would be helpful to continue to do so throughout. I also suggest moving the table that is currently in the supplement to the main document to help the reader understand and keep track of the various simulations.

Given the large number of simulations performed, we agree that these details can be challenging to track. To help improve clarity, we moved the table to the main text in the first subsection of the methods (which now precedes the results section), and added additional details such as a reference to which figures in the paper show the results for a given simulation.

Page 15: “muscle insertion points”

Comment: Do the authors mean “muscle attachment points”? (“insertion” refers only to the distal attachment).

Indeed, we mean attachment points and have updated the text accordingly. Thank you for pointing out this error.

Page 15: “Importantly, when scaling the skeleton, we chose to scale muscle volumes proportionally to the change in whole body mass and adjust body mass when scaling the body segments by assuming constant density. As such, a heavier model has stronger muscles.”

Comment: Please provide more detail on the scaling approach. This is a critical part of the study and it was difficult to fully understand from this brief statement.

We agree that the details of scaling are vital to the study design. We now provide more detail in the methods section (lines 145-148):

When scaling the skeleton, we chose to scale muscle volumes proportionally to the change in whole body mass with a 1:1 ratio. Individual body segments are scaled by assuming constant density. As such, the mass percentage of muscle and fat tissue remains constant for models of different sizes and thus a heavier model has stronger muscles.

Page 16 (Fig 4 caption): “where we turned the state-of-the-art from non-differentiable to differentiable computation”

Comment: Replace “state-of-the-art” with whatever was actually converted.

We updated the caption to make it more clear, based on this helpful feedback (lines 177-178):

The dotted boxes indicate the parts of the simulator where we turned non-differentiable computation used in OpenSim and Falisse et al. [40] into differentiable computation.

Page 18: “are bounded between -1 and 1 with 150 as in”

Comment: Should this be “150 N m”?

Yes indeed. We adapted accordingly (lines 217-218), “.…activations that are bounded between -1 and 1 with 150Nm as a scaling factor mapping torque actuator activation to torque .”

Page 18: “the talus, calcaneus, toes, shank, thigh, upper arms, lower arms, hands”

Comment: Consider replacing “calcaneus“ with something like “rear- and mid-foot” or explain that “calcaneus” does not actually mean just the calcaneus bone (this is due to an unfortunate naming scheme in some OpenSim models, similar to calling the shank segment the “tibia”).

Thank you for the suggestion; we have adapted the text accordingly by using the terms hind- and midfoot.

Page 19: “and as such changing the muscle maximal isometric force”

Comment: While p_V_muscle is applied to muscle volume it is described as changing maximal isometric force. As such, does it only affect the PCSA or does it also affect Lm_opt? Perhaps this could be explained better.

Whether Lm_opt is changed depends on whether the skeleton is scaled or not. In the strength simulations Lm_opt remains constant, and thus relative change in volume is identical to the relative change in PCSA. If the skeleton is scaled, lM_opt is adapted as explained in the Methods which is taken into account when adapting the PCSA when changing the overall volume.

We have made this more clear in the methods by adding the following sentence (line 250-252):

When the skeleton was scaled, V_muscle_  changed with a 1:1 ratio to the change in skeleton mass. In this case, PCSA could not be directly calculated from V_muscle, scaled_ as l_m,opt_  was first scaled to l_m,opt,scaled_ (see next paragraph).

Related comment: On page 23 there is a statement that seems relevant to this but is hard to understand “When simulating strength training, the increase of individual muscle physiological cross-sectional areas was limited to 20% and the total increase in muscle volume summed over all muscles was limited to 5% of the initial total muscle volume.” Please consider revising for clarity.

Thank you for pointing this out. We are now consistent in our wording and use muscle volume throughout the manuscript.

Page 19: “technical contribution of this work”

Comment: Change the wording so it is clear whether “this” refers to the present study or that of Falisse.

We changed the text to: “…  technical contribution of our work …” (line 267).

Page 20: “We chose the respective lower and upper limits of 0.8 and 1.2 as these cover most of the variation in an adult population.”

Comment: Please provide the reference that supports this statement.

We consulted the ANSUR II dataset, where we found that the range in anatomical measurements between the 1st and 99th percentile is around 14-17%. Our bounds are thus generous but realistic considering that ANSUR II is derived from a population of US Army personnel. Based on variation in the ANSUR II dataset, we chose the respective lower and upper limits of 0.8 and 1.2 as these cover most of the variation in an adult population [48].

We added a phrase in the methods to clarify (lines 294-296):

Based on variation in the ANSUR II dataset, we chose the respective lower and upper limits of   0.8 and 1.2 as these cover most of the variation in an adult population [47].

Page 21: “We put the model in that joint pose and observed the muscle tendon length and moment arm with respect to the joint coordinates that are actuated by the muscle.”

Comment: Given the large number of poses the model was placed in throughout the joint ROM, how did the authors handle the cases where (I assume) there would be some discontinuities in MT lengths and moment arms?

We added the following text to the manuscript to clarify how we handled discontinuities (lines 314-317):

The neural networks approximate the relationships in OpenSim between (1) joint coordinates and segment dimensions and (2) moment arms and muscles lengths. Discontinuities and non-smooth transitions in OpenSim moment arms and muscle lengths are smoothed because of the relatively shallow and small neural networks we used.

Page 21: “We then trained a separate neural network for each muscle using Adam [66] with a mean squared error loss over 1,000 epochs and a batch size of 64.”

Comment: Please briefly explain what “Adam” is and why it was selected.

We added a short explanation (Lines 304-305):

We then trained a separate neural network for each muscle using Adam [49], a stochastic gradient descent algorithm commonly used to optimize neural networks …”

Page 21: “With the described set-up we confirmed that our predictive simulations of walking and running at 3.33m/s yielded kinematics and muscle activation very close to the those with the original OpenSim model and for OpenSim models with all scaling factors at 0.85 or 1.15.”

Comment: I am confused by the reference to “our predictive simulations of walking” as there are no walking simulation results presented in this paper, which is about running. Please clarify.

We removed ‘walking’ from the phrase to avoid confusion. We initially tested for walking and later did some experiments to confirm for running as well.

Page 22: “we solved a trajectory optimization problem where either the skeleton scaling parameters (ps) or the scaling of muscle volumes (pVmuscle) were added to the optimization variables.”

Comment: Perhaps I missed it, but was there a reason to not include the case where both the skeleton and muscles were scaled in the same optimization? Was that computational prohibitive, or was there some other reason? If this is not already addressed then adding a brief statement seems appropriate.

This is certainly possible; we have updated our statement to be more accurate, since, in our framework, if the skeleton scaling parameters change, the muscle volumes also change. While there is nothing that prohibits optimizing both sets of parameters independently in the same problem, in this study, we chose to isolate, as much as possible, the effects of changing each. We added the following statement to help clarify these points (lines 344-345):

Note that if ps is optimized this directly determines pVmuscle as muscle volume is scaled with change in mass.

Page 22: “with Jmetabolic the muscle metabolic energy based on the Bhargava model [65] of metabolic energy expenditure”

Comment: In the paper by Falisse the metabolic energy rate was squared (their Eq 2.2). Was that term also squared in the present study?

Yes it was. We have updated the text to reflect this (line 350).

Page S2 (2nd page of the supplement): “The pelvis and lumbar degrees of freedom have similar waveforms”

Comment: This statement is not true for the pelvis rotation. The simulated data has nearly the opposite phasing compared with the experimental data.

We made this statement more accurate by explicitly noting that the pelvis rotation waveforms were not similar (lines 660-664).

Page S2: Figure S1

Comment: The figure on this page would be clearer if means and SDs bounds were plotted rather than the individual subject lines.

We chose to plot each individual experimental time-series as thin dotted grey lines alongside the simulated result to provide a comprehensive and nuanced depiction of the experimental data. We think, because there are only ten individual lines, displaying individual lines remains interpretable for the reader. When only showing mean and standard deviation it is unclear whether a standard deviation is the result of different curves having different shape around the mean or from different curves having the same shape but with an offset. A good example would be the pelvis posterior tilt, hip flexion, and lumbar extension angles where we can see that experimental variability mainly comes from offsets that we know can result from different marker placement, scaling and inverse kinematics. To allow the reader to see such nuances, we chose to keep the individual lines in the plots.

Page S3: “We compared our sprinting simulation to a sprinting gait cycle from an individual running at about 9.45m/s”

We added the simulation speed to the text (line 673; 8.13 m/s).

Reference section

Comment: The reference section is not up to typical standards. There are articles that appear more than once, there are references with information missing (e.g., missing journal name), and inconsistent formatting.

Thank you for pointing out these issues. We have made updates throughout the reference section accordingly.
